# Perceived Stress Predicts Subsequent Self-Reported Problems With Vision and Hearing: Longitudinal Findings From the German Ageing Survey

**DOI:** 10.1177/01640275211027304

**Published:** 2021-06-25

**Authors:** Markus Wettstein, Hans-Werner Wahl, Vera Heyl

**Affiliations:** 1 German Centre of Gerontology, Berlin, Germany; 2 Heidelberg University, Germany; 3 University of Education, Heidelberg, Germany

**Keywords:** sensory impairment, sensory abilities, midlife, old age, stressors, perceived stress scale

## Abstract

Although stress is a risk factor for various diseases in later life, its role for sensory abilities in the second half of life has rarely been empirically addressed. We examined if perceived stress at baseline predicts self-reported difficulties with vision and hearing 3 years later. We also explored whether chronological age is a moderator of associations between stress and sensory difficulties. Our sample was derived from the German Ageing Survey and consisted of *n* = 5,085 individuals aged 40–95 years (*M* = 64.01 years, *SD* = 10.84 years). Controlling for baseline self-reported sensory functioning, socio-demographic indicators, self-rated health and chronic diseases, greater perceived stress at baseline predicted greater self-reported difficulties with vision and hearing 3 years later. The effect of stress did not vary by age. Our findings suggest that, from middle adulthood to advanced old age, stress is a risk factor for increases in self-perceived problems with vision and hearing.

Sensory impairments are common in the second half of life, and the prevalence of—both objective and self-reported—impaired sensory functioning increases when individuals enter old and very old age (Bainbridge & Wallhagen, 2014; Homans et al., 2017; Horowitz et al., 2005; Keeffe, 2019; Leveziel et al., 2020; Peelle, 2020). The widespread negative consequences of sensory impairments for functional ability, quality of life and other psychosocial outcomes are well documented (Davis et al., 2016; Heyl & Wahl, 2014; Wettstein & Wahl, 2015). However, whether psychological factors may also function as —potentially modifiable—determinants of impaired vision and hearing in adulthood and old age, and not only as an outcome, is still largely unknown. Therefore, this study aims to examine the role of perceived stress as a predictor of subsequent self-reported difficulties with vision and hearing over 3 years in the second half of life. Moreover, the role of chronological age as a potential moderator of associations between stress and sensory problems, as well as the potentially mediating role of perceived stress on associations of depressive symptoms and self-perceptions of aging with subsequent hearing and vision problems will be examined. Doing so, we take advantage of a large study sample of adults aged between 40 and 95 years at baseline who were assessed twice within a 3-year observational period.

## Consequences of Impaired Vision and Hearing

Impaired vision and hearing pose a severe challenge on successful aging (Jang et al., 2003; Swenor et al., 2019; Wettstein et al., 2020) by affecting various developmental domains. Specifically, individuals with impaired sensory functioning have lower functional ability as well as a higher risk of disability than sensory unimpaired individuals (Chen et al., 2015; Liljas et al., 2016a). Sensory impaired individuals are also more often affected by compromised physical health and have a higher risk of incident morbidity across a broad range of different health conditions and even of mortality (Crews et al., 2017; Deal et al., 2019; Guilley et al., 2008; Liljas et al., 2016a, 2016b; Zimdars et al., 2012). Furthermore, several studies have found that poorer vision and hearing predict steeper decline in cognitive abilities (Liljas et al., 2018; Maharani et al., 2019; Swenor, Wang, et al., 2019) and are associated with a higher risk of cognitive impairments such as dementia (Albers et al., 2015; Davies et al., 2017; Deal et al., 2016; Fischer et al., 2016).

Finally, visual and hearing impairments are also associated with restricted psychosocial functioning, particularly with lower well-being, including greater depression (Cosh, Carriere, et al., 2018; Cosh, von Hanno, et al., 2018; Frank et al., 2019; Simning et al., 2018), anxiety (Cimarolli et al., 2016; Cosh, Naël, et al., 2018; Pinquart & Pfeiffer, 2011) and loneliness (Brunes et al., 2019; Shukla et al., 2020), lower affective well-being (Hajek et al., 2020; Heyl et al., 2007; Z. Liu et al., 2016), and reduced life satisfaction (Brown & Barrett, 2011; Good et al., 2008; Pinquart & Pfeiffer, 2011).

In conclusion, previous research has largely targeted visual and hearing impairment as antecedents of various developmental outcomes, with a detrimental impact of impaired sensory functioning on individuals’ health, cognitive abilities, and their quality of life. Interestingly, however, research examining psychological factors as *determinants*, rather than as outcomes, of sensory functioning, i.e. as factors that may either increase or reduce the risk of vision or hearing impairments has remained scarce.

## Psychological Determinants of Sensory Impairment in the Second Half of Life

Although research areas such as “psycho-ophthalmology,” “psychosomatic ophthalmology,” and “psycho-audiology”/“otogerontology” (Heyl & Wahl, 2014; Méndez-Ulrich & Sanz, 2017; Sabel et al., 2018; Wahl, 2013; Willott et al., 2001), which consider sensory impairment from a psychological perspective, are increasingly evolving, only a minority of studies has so far explicitly addressed psychological factors that may precede and predict impaired sensory functioning. Doing so may, however, open new perspectives on aspects of prevention and treatment of sensory impairments.

Notably, changes in vision and hearing across the second half of life, and particularly in later life, are remarkably heterogeneous (e.g., Wettstein et al., 2016; Whillans et al., 2016). However, factors beyond demographic or health-related indicators (Gerstorf et al., 2013; Hong et al., 2013; Wettstein et al., 2020; Whillans et al., 2016) that may affect sensory functioning and change thereof have rarely been empirically identified and investigated so far.

One exception in the vision domain is a study by Mueller et al. (2018), who found that a decrease in extraversion preceded late-life decline in close visual acuity. In young-old individuals only, increases in neuroticism also preceded subsequent decrease in visual acuity. In terms of hearing, Cosh, Naël, et al. (2018) found, based on 12-year longitudinal data, that greater anxiety was associated with a higher risk of self-reported hearing loss among older adults, whereas it did not predict objective near visual acuity. However, visual impairment as assessed by self-reports also seems to be predicted by anxiety and depression, as Frank et al. (2019) report based on a longitudinal study.

Additional evidence suggests that attitudes and mindsets (Langer et al., 2010) are also predictive of change in sensory abilities. Levy et al. (2006) found that older adults with more negative and more external age stereotypes showed a worse performance on an objective hearing test 3 years later than individuals who had more positive and less external age stereotypes. Notably, this effect was robust and remained statistically significant even when various socio-demographic and health-related variables were controlled for. In a recent study (Wettstein, Wahl, et al., 2020), this predictive effect of subjective aging on sensory functioning could be replicated, with a more favorable attitude toward own aging as well as higher age-related perceptions of continuous growth predicting less increase in self-reported problems with hearing over 9 years among older adults.

## The Role of Stress for Sensory Impairment

According to the established transactional stress model (Lazarus & Folkman, 1984), stress occurs when environmental demands exceed an individual’s resources (see also Aldwin et al., 2021). Stress is thus a subjective phenomenon, dependent on individual appraisals of a situation and of one’s personal resources.

Generally, “experiencing high levels of stress over an extended period of time is one of the biggest risk for mental and physical health” (Klaperski, 2018, p. 244). The detrimental impact of stress on health status and morbidity and the role of stress as a risk factor for various diseases and health symptoms (Aldwin et al., 2021; Almeida et al., 2002; Thoits, 2010), mental disorders (Charles et al., 2013), impaired cognitive functioning (McEwen, 2013; Neupert et al., 2006), and mortality (Aldwin et al., 2011; Jeong et al., 2016) is well established. Various pathways, including psychological as well as biological and physiological ones, seem to account for these health-detrimental effects of stress (Aldwin & Yancura, 2010; Kiecolt-Glaser et al., 2015; Stawski et al., 2013; Surachman & Almeida, 2018).

In addition, stress might also be a potential determinant of vision and hearing loss, which is in line with what previous stress-related research has found (Canlon et al., 2013; Muchnik et al., 1980; Sabel et al., 2018; Schmitt et al., 2000). For instance, according to Sabel et al. (2018), individuals with impaired vision often report that the onset of their vision loss was at a time of intense and prolonged stress. However, empirical evidence is still scarce and mostly based on cross-sectional study designs (Hasson et al., 2011)

With regard to mechanisms underlying the association between stress and sensory functioning, stress has been found to increase cytokines (Miller et al., 2002), which might have a detrimental impact both on vision (de Oliveira Dias et al., 2011; Sabel et al., 2018) and hearing (Adams, 2002). Also, stress is associated with elevated cortisol levels which have an impact on brain and sensory functioning via autonomous nervous system (sympathetic) imbalance, vascular dysregulation, and brain processing problems (Sabel et al., 2018). With regard to vision, stress affects the brain (Sapolsky, 1996), and “because the retina and eye are extensions of the brain (…), it may be conceivable that ‘ophthalmologic’ diseases might actually also be ‘brain’ diseases in disguise” (Sabel et al., 2018, p. 135). Hearing might be negatively affected by stress through effects on sympathetic stimulation of adrenergic α-receptors within the cochlea and on neuro-endocrine responses that engage the hypothalamic-pituitary-adrenal axis (Canlon et al., 2013).

In conclusion, prior research consistently suggests that stress might act as a risk factor for the onset or deterioration of problems with vision and hearing. However, there is still a lack of longitudinal investigations of the association between stress and sensory functioning as well as of studies examining both vision and hearing in parallel, as both sensory modalities might be differentially affected by stress. Also, as discussed in the next section, there is a lack of empirical approaches that consider stress as a mediating mechanism between psychosocial factors and sensory outcomes.

## Psychological Factors and Sensory Impairment: Stress as a Mediator

We argue that the previously described psychological predictors of impaired sensory functioning, such as indicators of mental health or of views on aging, do reveal one major commonality: They are all substantially linked with psychological stress and affect how individuals react and adjust to stressors.

Specifically, depression and anxiety may be caused by high levels of stress, as suggested, for instance, by the stress exposure model of depression (R. T. Liu & Alloy, 2010). However, associations are bi-directional, as depressive mood and depressogenic vulnerabilities also constitute and magnify stressors in people’s everyday life, thus causing a “stress generation effect” (Hammen, 2004; R. T. Liu & Alloy, 2010). Heightened stress could thus be one major underyling mechanism via which depressive symptoms and anxiety compromise sensory functioning (Cosh, Naël, et al., 2018; Frank et al., 2019; Kempen et al., 1996).

Finally, negative self-perceptions of aging may augment vulnerability to psychological distress (Losada-Baltar et al., 2020), and negative age stereotypes have been found to produce elevated physiological stress reactions, e.g. with regard to cardiovascular stress response (Levy et al., 2000, 2008), whereas positive age stereotypes might rather act as a “stress buffer” (Levy et al., 2016).

Concluding, stress may be a unifying mechanism underlying the impact of various psychological factors, such as depression or negative views on aging, on sensory functioning. We will address this assumption in our empirical analyses by specifying perceived stress as a mediator in the associations of depressive symptoms and self-perceptions of aging with subsequent sensory problems.

## Stress and Sensory Functioning: The Moderating Role of Age

As the consequences of stress in general might vary according to the specific phase of the life span when stress exposure occurred (Lupien et al., 2009), the role of stress for sensory functioning might as well not necessarily be the same across the entire second half of life, but change from middle adulthood to old age. Specifically, the model of strength and vulnerability integration (SAVI; Charles, 2010) states that there is an age-related increase in the use of strategies to avoid or limit the exposure to negative stimuli, and these negative stimuli might include stress-eliciting situations and daily hassles. At the same time, a major assumption of the SAVI model is that physiological flexibility declines with advancing age, so that negative situations eliciting high emotional arousal might have a stronger detrimental impact on individuals when they get older. Also, even in healthy older adults, the neuroendocrine and immune systems often show slower returns to normal after stress activation compared to younger persons (Aldwin, 2007). However, it should be taken into consideration that “knowing their greater vulnerability, older adults consciously avoid becoming upset by minor problems to prevent increases in health problems” (Aldwin & Yancura, 2010, p. 187).

To our knowledge, no study has so far investigated whether the effect of stress on sensory functions varies according to individuals’ chronological age. Given the theoretical considerations summarized above, an older age might, on the one hand, augment the “stress effect” on sensory functioning due to increasing physiological vulnerability among individuals who are older. On the other hand, older adults seem to avoid the occurrence of predictable stress situations and they might even be more skilled in using adequate coping strategies against stressors than younger adults (Aldwin et al., 2010; Brandtstädter, 2015; Diehl et al., 2014), thereby potentially also reducing the negative physiological consequences of stress, including those on sensory functioning. Given this ambiguity in theory and the lack of prior research, we will investigate the potentially moderating role of chronological age for the association between stress and sensory functioning in an exploratory way, without stating any specific a-priori hypothesis.

## Research Aims and Hypotheses

The aim of this study is to contribute to psycho-ophthalmology and psycho-audiology by investigating the role of stress as (1) a prospective predictor of problems with vision and hearing and (2) as a mediator of associations between psychological factors (depressive symptoms, self-perceptions of aging) and problems with vision and hearing. We expect that, controlling for relevant confounders (baseline vision/hearing problems, gender, age, region of residence, year of first study participation, education, self-rated health, and number of chronic diseases), greater perceived stress at baseline predicts greater self-reported difficulties with vision and hearing 3 years later. Given our age-heterogeneous study sample that comprises the entire second half of life from mid age to advanced old age, we also test at the exploratory level whether the association between perceived stress and subsequent problems with vision and hearing is moderated by chronological age.

In order to obtain additional insights regarding the question whether perceived stress has a stronger association with persistent sensory impairment than with sensory deterioration (or vice versa), we will also analyze in an exploratory manner how perceived stress is related with onset, persistence vs. improvement of self-reported sensory problems within 3 years.

Finally, based on our assumption that perceived stress is a mediator of associations between established psychosocial predictors and self-reported sensory functioning, we will also investigate if the data are in support of such a stress mediation model, with depressive symptoms and self-perceptions of aging being indirectly linked with subsequent self-reported sensory problems through perceived stress levels.

## Method

### Study Population and Sample Description

For our analyses, we used data of the German Ageing Survey (“Deutscher Alterssurvey,” DEAS). This survey is a cohort-sequential study, based on samples comprising community-dwelling adults aged 40 years and older at the time of their first study participation (Klaus et al., 2017; Vogel et al., 2020). Sample inclusion criteria were: aged between 40 and 85 years at study entry; living in a private household (i.e., not in institutions such as nursing homes) within Germany at the time of first study participation; ability to speak and understand German^1^.

If study participants consented, they were re-interviewed at the subsequent measurement occasions. Six measurement occasions (T1: 1996, T2: 2002, T3: 2008, T4: 2011, T5: 2014; T6: 2017) have been completed so far. Every 6 years (1996, 2002, 2008, 2014), a new sample was drawn based on national probability sampling. Samples were systematically stratified by age, gender, and region of residence (i.e., West or East Germany). Response rates of first-time participants between 1996 and 2014 were 50.3%, 37.8%, 35.7%, and 27.1%, respectively (Klaus & Engstler, 2017; Vogel et al., 2020).

For the present analyses, we included only study participants who took part at the measurement occasions in 2014 and 2017 because stress had not been assessed prior to 2014. Among those study participants whose first study participation was in 2014, re-participation rate in 2017 was 52% (Klaus et al., 2019), and among all individuals who participated in 2014, the re-participation rate was about 59%. Of those who participated both in 2014 and 2017 and who provided complete data on the study variables in 2014 (*n* = 5,168), 1.6% were excluded due to missing data at the follow-up measurement occasion, resulting in a final sample size of *n* = 5,085.^2^

A sample description is shown in Table 1. Participants’ mean age at baseline was 64.01 years (*SD* = 10.84 years). 50.7% of the sample were women. Intercorrelations between all study variables are shown in Supplemental Table 1. The frequencies of responses to all four items referring to self-reported problems with vision and hearing across both measurement occasions are shown in Supplemental Table 2.

**Table 1.** Sample Description.

	*M* ± *SD* or *n* (%)
Age (2014; 40–95 Years), *M* ± *SD*	64.01 ± 10.84
Female Sex, *n* (%)	2,578 (50.70%)
Education:	
Low, *n* (%)	254 (5.00%)
Medium, *n* (%)	2,544 (50.03%)
Elevated, n (%)	727 (14.30%)
High, *n* (%)	1,560 (30.68%)
Year of First Study Participation	
1996, *n* (%)	409 (8.04%)
2002, *n* (%)	521 (10.25%)
2008, *n* (%)	1,642 (32.29%)
2014, *n* (%)	2,513 (49.42%)
Region of Residence	
East Germany, *n* (%)	1,678 (33.00%)
Self-Rated Health^1^ (2014; 1–5), *M* ± *SD*	2.43 ± 0.80
Perceived Stress (2014; 1–5), *M* ± *SD*	2.32 ± 0.65
Perceived Stress (2017; 1–5), *M* ± *SD*	2.31 ± 0.64
Number of Chronic Diseases (2014; 0–11), *M* ± *SD*	2.51 ± 1.81
Depressive Symptoms (2014; 0–45), *M* ± *SD*	6.33 ± 5.77
Attitude Toward Own Aging (2014; 1–4), *M* ± *SD*	3.01 ± 0.53
Self-Reported Problems with Vision (Composite Score; 2014;1–4), M ± SD	1.15 ± 0.34
Self-Reported Problems with Vision (Composite Score; 2017; 1–4), M ± SD	1.17 ± 0.38
Self-Reported Problems with Hearing (Composite Score; 2014; 1–4), *M* ± *SD*	1.27 ± 0.47
Self-Reported Problems with Hearing (Composite Score; 2017;1–4), *M* ± *SD*	1.30 ± 0.50

*M* = mean; *SD* = standard deviation.

^1^ Lower values indicate better self-rated health.

### Measures

#### Self-reported problems with vision and hearing

Individuals reported their difficulties with vision and hearing across different contexts (Vision: “Do you have problems reading the newspaper [even when using a vision aid]?” and “Do you have problems recognizing familiar persons on the street [even when using a vision aid]?”; Hearing: “Do you have hearing problems with phone calls [even when using a hearing aid]?” and “Do you have problems with hearing in group meetings with four or more people [even when using a hearing aid]?”). They provided their responses on a 4-point Likert scale ranging from 1 (“no difficulties”) to 4 (“impossible”). Items with similar formulations have been used in other studies on visual and hearing impairment (e.g., Bourque et al., 2007; Frank et al., 2019; Horowitz et al., 1991; Liljas et al., 2016b; Xiang et al., 2019; Yip et al., 2014).

Both vision-related items and both hearing-related items revealed substantial intercorrelations (ranging between *r* = .44 and *r* = .61; see Supplemental Table 1). To increase the generality and reliability of our sensory outcomes, we aggregated the items reflecting vision and hearing problems. Specifically, we computed composite/mean scores of vision problems and hearing problems by averaging both vision items as well as both hearing items, resulting in a scale range of the composite scores from 1 to 4 (with possible values of 1, 1.5, 2, 2.5, 3, 3.5, and 4), with higher scores indicating more severe sensory problems.

#### Perceived stress

The 4-item short-form of the Perceived Stress Scale (S. Cohen et al., 1983) was used. The items ([1] “In the last month, how often have you felt that you were unable to control the important things in your life?”; [2] “In the last month, how often have you felt confident about your ability to handle your personal problems?”; [3] “In the last month, how often have you felt that things were going your way?”; [4]“In the last month, how often have you felt difficulties were piling so high that you could not overcome them?”) were answered on a 5-point Likert scale ranging from 1 = “never” to 5 = “very often” (Cronbach’s α = .70). A mean score of all items was computed, with higher scores corresponding to higher levels of perceived stress (items 2 and 3 were recoded accordingly).

#### Depressive symptoms

To explore the role of perceived stress as a mediator linking psychosocial factors and sensory problems, depressive symptoms were included as such a psychosocial factor in additional analyses. They were measured in 2014 using a 15-item German adaptation (Hautzinger & Bailer, 1993) of the Center for Epidemiological Studies Depression Scale (Radloff, 1977). Study participants were asked how often (on a 4-point scale ranging from “rarely or none of the time [less than 1 day]” to “most or all of the time [5–7 days]”) they had experienced 15 depressive symptoms during the last week (e.g., “I felt sad,” “My sleep was restless”). A sum score of all items was computed, with higher sum scores indicating more severe depressive symptoms (Cronbach’s α = .85).

#### Self-perceptions of aging

As additional psychosocial factor whose effect on sensory problems is potentially mediated by perceived stress, self-perceptions of aging were included. They were assessed in 2014 using the Attitude toward own aging (ATOA) subscale of the Philadelphia Geriatric Morale Scale (Lawton, 1975). This subscale comprises five items (e.g., “Things keep getting worse as I get older”) that are answered on a 4-point response format ranging from “strongly agree” to “strongly disagree.” A mean score was computed, with higher scores indicating a more favorable ATOA (Cronbach’s α: .72).

#### Covariates

We controlled for self-reported problems with vision/hearing in 2014 as well as for age, gender, education, year of first study participation (1996, 2002, 2008 or 2014), region of residence (West vs. East Germany), self-rated health and number of chronic diseases in our analyses. Education was assessed based on the International Standard Classification of Education (ISCED) coding of the UNESCO (2012). Specifically, four groups of school and professional education were differentiated (low, medium, elevated, and high education). Self-rated health was measured based on a single-item question (“How would you rate your current health?”). The response format ranged from 1 (very good) to 5 (very poor). Number of chronic diseases was assessed by a list comprising 11 chronic conditions (e.g., diabetes, cancer, cardiovascular diseases). Study participants had to indicate which of the listed illnesses they had. Responses were summed up, resulting in a sum score of chronic diseases for each individual.

### Statistical Analyses

Multiple linear regression models were computed with self-reported vision/hearing problems in 2017 specified as outcomes, and perceived stress in 2014 specified as predictor. We also included an age-stress interaction to explore if associations between stress and sensory problems are moderated by chronological age. For the regression analyses, age was mean-centered at 64.15 years, and perceived stress was centered by shifting its original range (1–4) to 0–3.

Additional predictors in the regression models were self-reported problems with vision/hearing in 2014 as well as age, gender, education, year of first study participation, region of residence, self-rated health and number of chronic diseases.

Moreover, we computed multinomial logistic regression models to examine how perceived stress is related to onset, persistence vs. improvement of self-reported sensory problems over time in comparison to individuals without sensory problems at both measurement occasions. Covariates included in these models were the same as in the multiple linear regression models (age, gender, education, year of first study participation, region of residence, self-rated health and number of chronic diseases).

**Figure 1.**
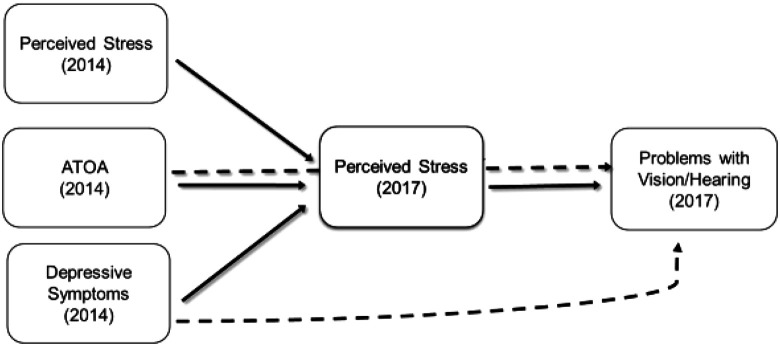
Illustration of the assumed stress mediation effect on associations of attitude toward own aging (ATOA) and depressive symptoms with sensory problem. *Note.* The effects of ATOA and depressive symptoms on problems with vision/hearing (dashed lines) are assumed to be partially mediated by perceived stress in 2017, controlling for perceived stress in 2014.

To test whether data are in support of a model specifying stress as a mediator of associations between psychosocial factors and sensory problems, we computed path models (see Figure 1), with sensory problems in 2017 predicted by depressive symptoms and ATOA in 2014 as well as by stress levels in 2017, controlling for self-reported problems with vision/hearing in 2014 as well as for all covariates described above. Additionally, in order to specify and test an indirect association of depressive symptoms and ATOA on sensory problems via perceived stress, we regressed perceived stress in 2017 on depressive symptoms and ATOA as well as on stress 2014.

## Results

### Stress as Predictor of Problems With Vision and Hearing

**Figure 2.**
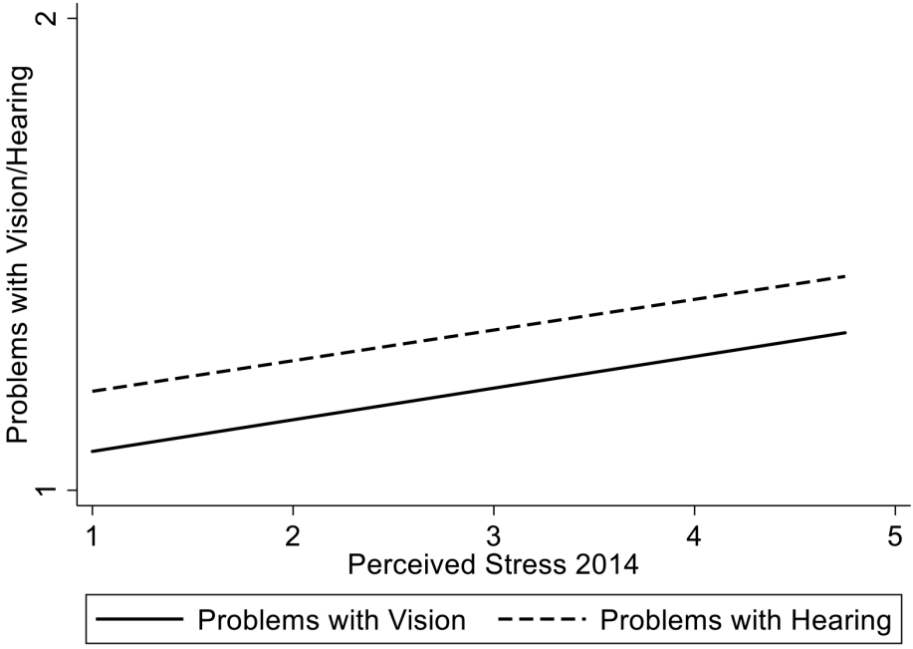
The association between stress (assessed in 2014) and perceived problems with vision and hearing (assessed in 2017; n = 5,085). *Note.* Higher scores on the y –axis indicate greater perceived difficulties with vision/hearing.

The results of the regression analyses are shown in Table 2. Amounts of variance accounted for by all predictors (R^2^) were .21 for vision problems and .42 for hearing problems. Controlling for baseline problems with vision/hearing, age, gender, education, region of residence, year of study entry, self-rated health and number of chronic diseases, higher stress in 2014 significantly predicted greater problems with vision and with hearing (see Figure 2). The standardized regression coefficient of stress was higher for vision (β = .04, *p* < .01) than for hearing (β = .02, *p* < .05).^3^ Regarding the role of chronological age, both stress × age interaction terms were not statistically significant, indicating that age did not significantly moderate the associations between stress and problems with vision or hearing.

**Table 2.** Predictors of Perceived Problems with Vision and Hearing (Assessed in 2017; n = 5,085).

Predictors	Problems With Vision (2017)		Problems With Hearing (2017)	
	B (SE)	β (SE)	B (SE)	β (SE)
Intercept	1.02***(0.02)		1.10*** (0.02)	
Problems with Vision (2014)	0.44*** (0.01)	.40*** (0.01)	—	—
Problems with Hearing (2014)	—	—	0.61*** (0.01)	.58*** (0.01)
Age (2014)	0.00 (0.00)	.04 (0.01)	0.01*** (0.00)	.12*** (0.01)
Self-Rated Health (2014)^a^	0.01* (0.01)	.03* (0.01)	−0.01 (0.01)	−.01 (0.01)
Chronic Diseases (2014)	0.02*** (0.00)	.08*** (0.01)	0.02*** (0.00)	.09*** (0.01)
Education	−0.00 (0.01)	−.01 (0.01)	0.00 (0.01)	.01 (0.01)
Gender^b^	0.02* (0.01)	.05* (0.03)	−0.04*** (0.01)	−.09*** (0.02)
Region of Residence^c^	0.01 (0.01)	.01 (0.03)	−0.03** (0.01)	−.06** (0.02)
First Study Participation^d^	−0.00 (0.01)	−0.01 (0.01)	−0.01 (0.01)	−.02 (0.01)
Stress (2014)	0.02** (0.01)	.04** (0.01)	0.02* (0.01)	.02* (0.01)
Stress (2014) × Age (2014)	0.00 (0.00)	.01 (0.01)	−0.00 (0.00)	−.02 (0.01)
R^2^ (total)	.21	.21	.42	.42

*Note.* B = unstandardized regression coefficients, β = standardized regression coefficients. SE = standard error.

^a^ Higher values indicate poorer self-rated health. ^b^ 0 = male, 1 = female. ^c^ 0 = West Germany, 1 = East Germany. ^d^0 = 1996, 1 = 2002, 2 = 2008, 3 = 2014.

* *p* < .05; ** *p* < .01; *** *p* < .001.

### Perceived Stress and Onset, Persistence vs. Improvement of Sensory Problems

For the multinomial logistic regression analyss with vision problems as outcome, we first differentiated between individuals with no vision problems at both measurement occasions (stable visually unimpaired group/ reference group; *n* = 3,386; 66.6%), individuals who reported no vision problems in 2014, but at least some vision problems in 2017 (vision deterioration group; *n* = 660; 13.0%), persons with at least some vision problems in 2014, but no vision problems in 2017 (vision improvement group; *n* = 526; 10.3%), and individuals who had at least some vision problems both in 2014 and 2017 (stable visually impaired group; *n* = 513; 10.1%). Controlling for age, gender, education, year of first study participation, region of residence, self-rated health and number of chronic diseases, higher perceived stress was associated with a higher risk of belonging to the vision deterioration group (relative risk ratio = 1.17, SE = 0.08, *p* = .03), as well as with a higher risk of belonging to the vision improvement group (relative risk ratio = 1.31, SE = 0.10, *p* < .001) and to the stable visually impaired group (relative risk ratio = 1.49, SE = 0.12, *p* < .001) than to the reference group of stable visually unimpaired individuals.

Next, we applied the same differentiation to hearing problems, distinguishing between stable hearing-unimpaired individuals (reference group; *n* = 2,965; 58.3%), individuals with no self-reported hearing problems in 2014, but at least some hearing problems in 2017 (hearing deterioration group; *n* = 492; 9.7%), persons who reported at least some hearing problems in 2014, but no longer in 2017 (hearing improvement group; *n* = 403; 7.9%), and those who reported at least some hearing problems at both occasions (stable hearing-impaired group*; n* = 1,224; 24.1%). Multinomial logistic regression analyses revealed that—again controlling for age, gender, education, year of first study participation, region of residence, self-rated health and number of chronic diseases—greater perceived stress was only related to a greater risk of belonging to the stable hearing-impaired group than to the stable hearing–unimpaired group (relative risk ratio = 1.32, SE = 0.08, p < .001). This effect was additionally moderated by chronological age, with the effect of perceived stress on the risk of belonging to the stable hearing-impaired group decreasing with advancing age (relative risk ratio _stresss × age_ = 0.98, SE = 0.01, *p* < .001). The risk of belonging to any other group in comparison to the reference group of stable hearing-unimpaired individuals was not significantly predicted by perceived stress.

### Perceived Stress as a Mediator of the Effect of Depressive Symptoms and ATOA on Subsequent Sensory Problems

We specified path models, as illustrated by Figure 1 predicting problems with vision/hearing in 2017 by depressive symptoms and ATOA in 2014 and by perceived stress in 2017 (controlling for self-reported problems with vision/hearing in 2014 as well as age, gender, education, year of first study participation, region of residence, self-rated health and number of chronic diseases). Additionally, to test if there is an indirect effect of ATOA and depressive symptoms on sensory problems, paths were specified leading from ATOA, depressive symptoms and stress in 2014 to stress in 2017 (controlling for stress in 2014).

The mediation model with vision problems as outcome revealed, according to conventional criteria (e.g., Hu & Bentler, 1999), a very good model fit (χ^2^(9) = 76.64, *p* < .001; CFI = .976; RMSEA = .039, *p* = .99; R^2^_vision problems__2017_ = .21, *p* < .001). Results were not in support of our mediation assumption. Specifically, higher depressive symptoms and less favorable ATOA were directly associated with more subsequent vision problems (β _depressive symptoms_ = .04, *p* = .01; β _ATOA_ = −.04, *p* = .02), but as perceived stress, assessed in 2017, was not a significant predictor of vision problems (β _stress_ = .02, *p* = .19), there was thus no significant indirect association of depressive symptoms and ATOA on vision problems via perceived stress.

With regard to hearing problems, the mediation model also revealed a very good model fit (χ^2^(9) = 76.19, *p* < .001; CFI = .986; RMSEA = .039, *p* = .99; R^2^_hearing problems__2017_ = .42, *p* < .001). Depressive symptoms and ATOA were not significantly directly related with problems with hearing (β _depressive symptoms_ = .00, *p* = .90; β _ATOA_ = −.02, *p* = .16), but estimates were in support of a mediation model of indirect associations, with significant effects of higher depressive symptoms and less favorable ATOA on higher perceived stress in 2017 (β _depressive symptoms_ = .07, *p* < .001; β _ATOA_ = −.15, *p* < .001), which was in turn significantly associated with greater hearing problems in 2017 (β _stress_ = .04, *p* = .002).

## Discussion

In this study, we investigated the role of perceived stress for subsequent self-reported problems with vision and hearing over a 3-year time span among individuals aged 40–95 years. We also investigated if the association of perceived stress with these sensory outcomes varies by chronological age and if associations between psychosocial factors (depressive symptoms and self-perceptions of aging), perceived stress and sensory problems are consistent with a mediation model specifying stress as mechanism underlying the association between these psychosocial components and problems with vision or hearing.

### Stress and Subsequent Problems With Vision and Hearing

We were able to demonstrate in a large, age-heterogeneous sample of middle-aged, older, and very old individuals that greater perceived stress is indeed a prospective predictor of more problems with both vision and hearing 3 years later. Notably, this predictor effect of stress was robust and significant even when controlling for baseline sensory problems, self-rated health, number of chronic diseases, as well as sociodemographic characteristics. When additionally differentiating between problems with near vision vs. with distant vision, we found a robust and consistent effect of perceived stress on both vision components. As also suggested by others (Canlon et al., 2013; Muchnik et al., 1980; Sabel et al., 2018; Schmitt et al., 2000) stress thus seems to be a determinant of sensory functioning, although prior research has mostly considered stress as an outcome of sensory impairments.

With regard to other studies that investigated and identified psychosocial predictors of vision and hearing, such as neuroticism, anxiety, depression, or views on aging (Cosh, Naël, et al., 2018; Frank et al., 2019; Kempen et al., 1996; Levy et al., 2006; Mueller et al., 2018; Wettstein, Wahl, et al., 2020), stress might be the mediating mechanism, as all these predictors are associated with stress (Hammen, 2004; Levy et al., 2000, 2008, 2016; R. T. Liu & Alloy, 2010). Therefore, stress deserves more empirical and conceptual attention in psycho-ophthalmological and psycho-audiological research as it might play a key role for vision and hearing problems, potentially representing a crucial mediating mechanism linkingn various psychosocial factors and sensory outcomes. Although we were not able to test this assumption for all these psychosocial factors, as we had, for instance, no measure of neuroticism available, findings of additional mediation analyses were indeed in support of a mediating role of perceived stress on associations of depressive symptoms and ATOA with subsequent hearing problems (but not with subsequent vision problems). Depressive symptoms and ATOA thus possibly exert a “stress generation effect” (Hammen, 2004; R. T. Liu & Alloy, 2010) that in turn affects sensory functioning. However, this finding is not a “proof” of mediation, and the associations observed are also in support of various alternative models, which is a main limitation of mediation analyses in general (see Fiedler et al., 2018). Also, no mediating role of perceived stress was found for vision problems so that such stress mediation effects are probably limited to specific sensory modalities as well as to certain psychosocial resources. Associations might be even more complex, including both a stress exposure effect on depression as well as a stress generation effect by depressive symptoms, which requires “moving away from unidirectional models of the stress-depression association” and consideration of “progressive and dynamic relationships between stress and depression over time” by future research (Hammen, 2004, p. 293). This is also true with regard to the associations between perceived stress and self-reported problems with sensory functioning in general, which we investigated unidirectionally, focusing on the so far neglected role of stress as a risk factor for sensory impairment. However, associations might be more complex and reciprocal, with stress and sensory problems reinforcing each other, potentially resulting in a “vicious cycle of a downward spiral” (Sabel et al., 2018, p. 133) of continuous increase in stress and decrease in sensory functioning. Such complex, potentially bi-directional and dynamic associations need to be further investigated by future studies.

In terms of effect sizes, the predictive contribution of stress might be considered as small. However, is it important to point out that the effects reported indicate the unique predictive impact of stress when controlling for various factors, including self-rated health, multimorbidity/number of chronic diseases, baseline sensory problems, and socio-demographic characteristics. Moreover, comparing the relative impact of the included predictors, the standardized regression coefficient of stress as predictor of vision problems was similar in size as the one of self-rated health. Stress was also a significant predictor of hearing problems, whereas self-rated health was not. Moreover, stress seems to be a more important determinant of subsequent problems with vision and hearing than education, which was not systematically related with these problems.

We also found, based on multinomial logistic regression models, that greater perceived stress predicted a higher risk of onset of vision problems and persisting vision problems in comparison to no vision problems at both measurement occasions. Greater perceived stress was also associated with a higher risk of persisting hearing problems in comparison to no hearing problems over 3 years. Stress thus seems to be associated with both onset and persistence of vision problems, with a stronger effect on the persistence of these problems, whereas it is associated with the persistence, rather than the onset, of hearing problems. Surprisingly, greater perceived stress was also associated with a higher risk of improvement of vision problems over time compared to no vision problems at both occasions. This effect could indicate that high levels of stress at one point in time might cause a transitory onset of vision problems which disappear once stress levels decrease. Alternatively, some individuals might perceive high levels of stress due to the onset of vision problems, and these heightened stress levels might motivate them to seek optimal treatment or compensation for sensory loss, resulting in less severe vision problems 3 years later. In this study, we do not have detailed information regarding underlying eye diseases available, and we do not know which individuals had specific treatments (such as glaucoma surgery) between both measurement occasions. More research is therefore needed to investigate the role of stress for both deterioration of and recovery from sensory impairment.

Our findings might have important practical implications. Interventions to reduce stress or to promote the use of adaptive coping strategies when facing and dealing with stressors might help to reduce the various health risks that stress conveys (Aldwin et al., 2021; Almeida et al., 2002; Thoits, 2010), including the risk of impaired vision or hearing. Different coping strategies, such as flexible goal adjustment, have already been found to be associated with higher well-being among sensory impaired older adults (Boerner & Wang, 2012; Brennan-Ing et al., 2013; Heyl et al., 2007; Wettstein et al., 2019), and these strategies might also contribute to preventing the onset or progression of sensory deficits by avoiding or reducing stress and by buffering the detrimental effect of stress. Treatment and prevention of sensory impairment might thus benefit from taking a psycho-ophthalmological and psycho-audiological perspective by not only focusing on medical and biological risk factors for vision and hearing loss, but also on psychological factors that might contribute to the onset or progression of sensory impairments.

Due to large interindividual differences with regard to stress-relevant factors such as stress sensitivity, stress reactivity, coping strategy use or resilience (Almeida, 2005; Charles et al., 2013; Mroczek & Almeida, 2004), not all individuals can be expected to experience to the same extent negative health consequences, including sensory problems, due to stress (Aldwin & Yancura, 2010). Future research should therefore investigate the role of potential moderators that might modulate the negative impact of stress on sensory problems, in line with the central assumption of established diathesis-stress models that certain factors can either increase or reduce an individual’s general vulnerability to stress (Hammen, 2004).

### The Role of Age

The predictor effect of stress on both vision and hearing problems was not moderated by chronological age. Our findings thus imply that throughout the second half of life, stress plays a detrimental role for problems with vision and hearing, but stress does neither become more nor less detrimental for these sensory problems with advancing age. General age-related trends of increase in both physical vulnerability (Baltes & Smith, 2003; Charles, 2010) and adaptive coping (Diehl et al., 2014) could thus neutralize each other, so that stress does not necessarily become more detrimental or less detrimental with advancing age. Alternatively, given the remarkable “aged heterogeneity” (Nelson & Dannefer, 1992) among older adults, and also in line with common diathesis-stress models, there may be some “resource-rich” (Lang et al., 2002) older individuals who have gained more and more coping expertise with increasing life time and thus have sufficient coping strategies, self-regulatory potential as well as other resources available to avoid stressors and to reduce their detrimental consequences. In contrast, other older adults who are more vulnerable and rather “resource-poor” might be more sensitive to stress and its negative consequences, including those on sensory functioning.

### Limitations

It is important to point out that the study outcome variables were *self-reported* problems with vision and hearing rather than objective sensory functions, and there is, of course, no perfect correlation between both assessment modes. Some individuals perform well on objective sensory tests, but complain about sensory problems in everyday life. Others reveal objective sensory deficits, but they do not report severe subjective problems with sensory functioning. Stress might cause more negative self-perceptions of one’s sensory abilities without affecting objective sensory functioning (Kim et al., 2017). However, stress generally seems to have a stronger impact on objective health outcomes than on indicators of subjective health (Aldwin, 2007), and objective sensory loss is not always mirrored by self-reported sensory loss of older adults (Bainbridge & Wallhagen, 2014; Choi et al., 2015). Further research is thus needed to contrast potential differences in stress effects on objective vs. subjective sensory outcomes. Specifically, the potential biological mechanisms linking stress and sensory functioning which we discussed in the introduction, but which we were not able to investigate in this study, might require assessment of objective, rather than subjective sensory impairments.

However, regarding the differences between objective and subjective sensory functioning, there are also studies demonstrating that subjective and objective sensory abilities are remarkably congruent (Cavazzana et al., 2018; Nondahl et al., 1998; Sindhusake et al., 2001). For instance, according to Whillans and Nazroo (2014), 97% with self-reported normal vision also score normal on an objective vision test. Similarly, a concordance rate of 75% between objective and self-reported vision was reported by Pinto et al. (2014). The concordance rates for hearing seem to be in a similar range (Kim et al., 2017), so that assessing hearing loss based on a self-reported single-item assessment “cannot substitute audiometry, but it can assess hearing loss on a population level with reasonable accuracy” (Oosterloo et al., 2020). Therefore, subjective measures of sensory functioning similar to the ones used in this study are often included in large-scale studies whenever objective assessment is not possible.

Generally, the proportion of individuals with severe vision or hearing problems was very low in our non-clinical study sample. It is likely that, given the overall low response rate, among individuals with intact vision and hearing, participation rate was higher than among those with sensory impairments. Also, it can be assumed that more individuals whose sensory abilities and sensory problems deteriorated dropped out of the study after 2014 compared to those with more stable sensory functioning, though our sample selectivity analyses with regard to all study variables assessed in 2014 indicated that selectivity effects were consistently of small effect size. Given the overall somewhat restricted range in vision and hearing problems and in 3-year changes thereof, stronger effects of stress might have been observed if more individuals with severe sensory problems or whose vision or hearing problems considerably deteriorated over 3 years had been included in the sample, or if the observation period had been longer than 3 years. The question to which extent *severe* sensory impairments further progress when individuals are affected by high stress levels, or maybe get better after longer “stress-free” periods, can therefore not be sufficiently addressed based on our data and requires further investigation.

Finally, our measure of perceived stress, though being an established and frequently used assessment instrument with good psychometric properties, is limited in that it cannot distinguish between acute and chronic stress, with the latter one having a stronger detrimental impact on health outcomes (Schneiderman et al., 2005).

## Conclusion

Our findings suggest that sensory impairments may not be exclusively caused by biological and physiological factors such as age-related degradations of organic systems which are not easily reversible. Rather, stress as a psychosocial factor plays a unique role in the prediction of the occurrence and progression of sensory problems. Therefore, stress deserves more attention with regard to its role for prevention and treatment of sensory impairments. Interventions aiming at stress prevention or at improvement of stress management, e.g. by use of adequate coping strategies, might be a promising contribution to promote not only “vision health” and “hearing health” (Davis et al., 2016; Russ et al., 2018), but in consequence also quality of life and autonomy in middle-aged and older adults.

## Supplemental Material

Supplemental Material, sj-docx-1-roa-10.1177_01640275211027304 - Perceived Stress Predicts Subsequent Self-Reported Problems With Vision and Hearing: Longitudinal Findings From the German Ageing Survey Click here for additional data file. Supplemental Material, sj-docx-1-roa-10.1177_01640275211027304 for Perceived Stress Predicts Subsequent Self-Reported Problems With Vision and Hearing: Longitudinal Findings From the German Ageing Survey by Markus Wettstein, Hans-Werner Wahl and Vera Heyl in Research on Aging

## Notes

1 At the measurement occasions prior to 2008, German citizenship was an inclusion criterion. However, from 2008 on, also Non-Germans could take part in the survey as long as they were living in Germany and had German language proficiency (Klaus, Engstler, & Vogel, 2019). In our study sample, 1.1% (*n* = 55) had a non-German citizenship (e.g., Turkey, Spain, France, Ukraine), and 4.1% (*n* = 210) had a migration background.

2 We conducted analyses of sample selectivity by comparing all study variables (assessed in 2014) between those individuals who took part both in 2014 and in 2017 and those who were lost to follow-up after 2014. Regarding continuous variables, only the group difference for self-reported difficulties with hearing in group meetings was not significant. All other differences were statistically significant, with those lost to follow-up being, on average, older, revealing a poorer self-rated health, higher levels of perceived stress, more chronic diseases and more self-reported problems with vision and hearing than those who re-participated in the study in 2017. However, the effect sizes of all differences were—according to common effect size classifications (J. Cohen, 1992)—consistently small, ranging between *d* = .06 (self-reported problems with phone calls) and *d* = .28 (ATOA). With regard to categorical variables, the distributions of gender and region of residence were not significantly different between groups, but there was a significantly higher proportion of low education in individuals lost to follow up than in those who remained in the study (Cramer’s V = .12). Also, the proportion of individuals with first study participation in 2014 was larger in the “lost-to follow-up” group than in the group of re-participants (Cramer’s V = .18).

3 As problems with near vision (reading the newspaper) might be more “age-normative,” corresponding to the common phenomenon of presbyopia, and might result from a different etiology than problems with distant vision (recognizing persons on the street), we considered these two items as separate outcomes in additional regression analyses. Greater perceived stress was significantly associated with both outcomes (problems reading the newspaper: β _stress_ = .05, *p* = .001; problems recognizing persons on the street: β _stress_ = .03, *p* = .02).
