# Supplementary material for: Perceived Stress Predicts Subsequent Self-Reported Problems With Vision and Hearing: Longitudinal Findings From the German Ageing Survey
Source: Res Aging. 2021 Jun 25;44(3-4):286–300. doi: 10.1177/01640275211027304 (PMC8948369; doi:10.1177/01640275211027304)
Supplement: Supplemental Material, sj-docx-1-roa-10.1177_01640275211027304 - Perceived Stress Predicts Subsequent Self-Reported Problems With Vision and Hearing: Longitudinal Findings From the German Ageing Survey [file sj-docx-1-roa-10.1177_01640275211027304.docx]

Supplemental Table 1

*Correlations Between Study Variables*

|  |  |  | |  | |  | |  | |  |  |  |  |  |  |  |  |  |  |  |  |  |  |  |  |  |
| --- | --- | --- | --- | --- | --- | --- | --- | --- | --- | --- | --- | --- | --- | --- | --- | --- | --- | --- | --- | --- | --- | --- | --- | --- | --- | --- |
|  | 1  Age | 2  Sex ^a^ | | 3  Education | | 4  Study Entry ^b^ | | 5  Region ^c^ | | 6  Self-Rated Health 2014 ^d^ | 7  Perceived Stress 2014 | 8  Chronic Diseases 2014 | 9 Problems Newspa-per 2014 | 10  Problems Newspa-per 2017 | 11  Problems Recog-nizing Persons 2014 | 12  Problems Recog-nizing Persons 2017 | 13  Problems Phone Calls 2014 | 14  Problems Phone Calls 2017 | 15  Problems Hearing in Groups 2014 | 16  Problems Hearing in Groups 2017 | 17  Vision Com-posite 2014 | 18  Vision Com-posite 2017 | 19 Hearing Com-posite 2014 | 20 Hearing Com-posite 2017 | 21  ATOA 2014 | 22 Depres-sive Symp-toms 2014 |
| 1 | 1 | -.094** | | -.053** | | -.236** | | .018 | | .082** | -.061** | .313** | .060** | .084** | .087** | .118** | .189** | .217** | .237** | .231** | .084** | .115** | .242** | .250** | -.103** | -.062** |
| 2 |  | 1 | | -.186** | | -.030* | | .010 | | -.015 | .066** | -.032* | .037** | .028* | .032* | .046** | -.040** | -.061** | -.097** | -.115** | .041** | .040** | -.082** | -.102** | .022 | .090** |
| 3 |  |  | | 1 | | .034* | | .079** | | -.117** | -.165** | -.085** | -.020 | -.043** | -.007 | -.031* | -.053** | -.048** | -.005 | .010 | -.018 | -.044** | -.029* | -.017 | .128** | -.101** |
| 4 |  |  | |  | | 1 | | -.021 | | -.010 | .020 | -.058** | -.005 | -.019 | -.014 | -.041** | -.043** | -.053** | -.037** | -.058** | -.010 | -.034* | -.044** | -.062** | .012 | .045* |
| 5 |  |  | |  | |  | | 1 | | .071** | .009 | .035* | .021 | .034* | .040** | .009 | .002 | -.007 | -.050** | -.064** | .033* | .026 | -.031* | -.044** | -.065** | .039** |
| 6 |  |  | |  | |  | |  | | 1 | .305** | .415** | .114** | .122** | .084** | .101** | .097** | .093** | .110** | .094** | .119** | .131** | .117** | .104** | -.417** | .442** |
| 7 |  |  | |  | |  | |  | |  | 1 | .225** | .109** | .110** | .078** | .085** | .065** | .075** | .093** | .076** | .113** | .114** | .009** | .084** | -.483** | .498** |
| 8 |  |  | |  | |  | |  | |  |  | 1 | .177** | .179** | .145** | .162** | .270** | .276** | .317** | .277** | .192** | .198** | .332** | .308** | -.329** | .288** |
| 9 |  |  | |  | |  | |  | |  |  |  | 1 | .354** | .443** | .276** | .165** | .078** | .137** | .104** | .897** | .369** | .167** | .103** | -.113** | .146** |
| 10 |  |  | |  | |  | |  | |  |  |  |  | 1 | .252** | .489** | .096** | .156** | .125** | .156** | .365** | .900** | .126** | .174** | -.141** | .154** |
| 11 |  |  | |  | |  | |  | |  |  |  |  |  | 1 | .402** | .178** | .087** | .172** | .095** | .795** | .365** | .196** | .102** | -.076** | .080** |
| 12 |  |  | |  | |  | |  | |  |  |  |  |  |  | 1 | .096** | .179** | .124** | .158** | .386** | .820** | .125** | .185** | -.100** | .087** |
| 13 |  |  | |  | |  | |  | |  |  |  |  |  |  |  | 1 | .487** | .589** | .434** | .201** | .112** | .855** | .508** | -.093** | .095** |
| 14 |  |  | |  | |  | |  | |  |  |  |  |  |  |  |  | 1 | .453** | .608** | .095** | .194** | .523** | .865* | -.105** | -094** |
| 15 |  |  | |  | |  | |  | |  |  |  |  |  |  |  |  |  | 1 | .619** | .179** | .145** | .923** | .609** | -.112** | .108** |
| 16 |  |  | |  | |  | |  | |  |  |  |  |  |  |  |  |  |  | 1 | .118** | .182** | .604** | .925** | -.122** | .076** |
| 17 |  |  | |  | |  | |  | |  |  |  |  |  |  |  |  |  |  |  | 1 | .431** | .210** | .120** | -.114** | .139** |
| 18 |  |  | |  | |  | |  | |  |  |  |  |  |  |  |  |  |  |  |  | 1 | .146** | .208** | -.142** | .143** |
| 19 |  |  | |  | |  | |  | |  |  |  |  |  |  |  |  |  |  |  |  |  | 1 | .633** | -.116** | .114** |
| 20 |  |  | |  | |  | |  | |  |  |  |  |  |  |  |  |  |  |  |  |  |  | 1 | -.127** | .094** |
| 21 |  |  | |  | |  | |  | |  |  |  |  |  |  |  |  |  |  |  |  |  |  |  | 1 | -.441** |
| 22 |  |  | |  | |  | |  | |  |  |  |  |  |  |  |  |  |  |  |  |  |  |  |  | 1 |
|  |  | |  | |  | |  | |  |  |  |  |  |  |  |  |  |  |  |  |  |  |  |  |  |  |

*Note.* ** *p* < .01; *** *p* < .001. ATOA = attitude toward own aging.

a 0 = male, 1 = female.

b 0 = 1996, 1 = 2002, 2 = 2008, 3 = 2014

c 0 = West Germany, 1 = East Germany.

d Higher values indicate poorer self-rated health.

Supplemental Table 2

*Self-Reported Problems with Vision and Hearing (2014 and 2017)*

|  | | *n* (%) |
| --- | --- | --- |
| **Self-Reported Problems with Vision and Hearing:** |  | |
| Problems Reading the Newspaper (2014) |  | |
| No Difficulties, *n* (%)  Some Difficulties, *n* (%)  Major Difficulties, *n* (%)  Impossible, *n* (%) | 4,184 (82.30%)  781 (15.36%)  108 (2.12%)  11 (0.22%) | |
| Problems Reading the Newspaper (2017), *M*±*SD* |  | |
| No Difficulties, *n* (%)  Some Difficulties, *n* (%)  Major Difficulties, *n* (%)  Impossible, *n* (%) | 4,071 (80.06%)  878 (17.27%)  114 (2.24%)  22 (0.43%) | |
| Problems Recognizing Familiar Persons on Street (2014) |  | |
| No Difficulties, *n* (%)  Some Difficulties, *n* (%)  Major Difficulties, *n* (%)  Impossible, *n* (%) | 4,706 (92.60%)  313 (6.16%)  51 (1.00%)  12 (0.24%) | |
| Problems Recognizing Familiar Persons on Street (2017) |  | |
| No Difficulties, *n* (%)  Some Difficulties, *n* (%)  Major Difficulties, *n* (%)  Impossible, *n* (%) | 4,611 (90.71%)  397 (7.81%)  55 (1.10%)  19 (0.37%) | |
| Problems With Phone Calls (2014) |  | |
| No Difficulties, *n* (%)  Some Difficulties, *n* (%)  Major Difficulties, *n* (%)  Impossible, *n* (%) | 4,281 (84.20%)  690 (13.60%)  103 (2.00%)  9 (0.20%) | |
| Problems With Phone Calls (2017) |  | |
| No Difficulties, *n* (%)  Some Difficulties, *n* (%)  Major Difficulties, *n* (%)  Impossible, *n* (%) | 4,192 (82.50%)  748 (14.70%)  130 (2.60%)  13 (0.30%) | |
| Problems With Hearing in Group Meetings (2014) |  | |
| No Difficulties, *n* (%)  Some Difficulties, *n* (%)  Major Difficulties, *n* (%)  Impossible, *n* (%) | 3,600 (70.80%)  1,159 (22.80%)  308 (6.10%)  12 (0.20%) | |
| Problems With Hearing in Group Meetings (2017) |  | |
| No Difficulties, *n* (%)  Some Difficulties, *n* (%)  Major Difficulties, *n* (%)  Impossible, *n* (%) | 3,520 (69.20%)  1,181 (23.20%)  357 (7.00%)  20 (0.40%) | |
